# A prevalence study of COVID-19 among healthcare workers in a pandemic hospital in the Samsun province of Turkey

**DOI:** 10.1371/journal.pone.0279067

**Published:** 2022-12-22

**Authors:** Mehmet Hakan Taskin, Zafer Yazici, Gerald Barry

**Affiliations:** 1 Department of Medical Microbiology, Samsun Training and Research Hospital, University of Health Sciences, Samsun, Turkey; 2 Department of Virology, Faculty of Veterinary Medicine, Ondokuz Mayis University, Samsun, Turkey; 3 School of Veterinary Medicine, University College Dublin, Dublin, Ireland; Imam Abdulrahman Bin Faisal University, SAUDI ARABIA

## Abstract

Among populations globally, many healthcare workers have been disproportionally impacted by the COVID-19 pandemic because of their above average exposure to people infected with SARS-CoV-2. Exposure to asymptomatic or pre-symptomatic individuals is particularly challenging, if those individuals continue to work, not knowing that they are potentially infectious. This study aimed to measure the level of asymptomatic infection in a cohort of workers in a healthcare setting in Turkey during the second major wave of infection in late 2020. Blood samples were collected and tested by electrochemiluminescence immunoassay for SARS-CoV-2 IgM and IgG antibodies. Nasal and throat swabs were performed in a subset of this cohort and RT-qPCR was used to search for the presence of SARS-CoV-2 RNA. The results showed that approximately 23% of the cohort were positive for anti-SARS-CoV-2 IgM antibodies and approximately 22% were positive for anti-SARS-CoV-2 IgG antibodies despite no reported history of COVID-19 symptoms. Just less than 30% of a subset of the group were positive for the presence of SARS-CoV-2 RNA indicating the likelihood of a current or recent infection, again despite a lack of typical COVID-19 associated symptoms. This study indicates a high rate of asymptomatic infection and highlights the need for regular testing of groups such as healthcare workers when community prevalence of disease is high and there is a desire to limit entry of virus into settings where vulnerable people may be present, because symptoms cannot be relied on as indicators of infection or infectiousness.

## Introduction

Cases of Coronavirus Disease-2019 (COVID-19) began to emerge in the Wuhan province of China at the end of 2019, causing severe pneumonia with unknown aetiology. It rapidly spread to other provinces in China and beyond to the rest of the world. The cause of this disease was first identified as a novel coronavirus by the Chinese Centre for Disease Control in early January 2020 and The World Health Organisation (WHO) declared the situation to be a public health emergency in China in mid-January 2020. The name of the new virus, severe acute respiratory syndrome coronavirus-2 (SARS-CoV-2), was first announced by the International Committee on Taxonomy of Viruses (ICTV) in February 2020 [1] and around the same time, the WHO announced the term COVID-19 to describe the disease caused by SARS-CoV-2. On the 11th of March, 2020, The WHO declared that COVID-19 was a global pandemic.

SARS-CoV-2 is an RNA virus in the family *Coronaviridae*, genus *Betacoronavirus*, and subgenus *Sarbecovirus*. Phylogenetic analysis has placed SARS-CoV-2 in the same cluster as bat-SARS-like coronaviruses, sharing approximately 96% nucleotide identity (96.8% for BANAL-52 and 96.1% for RaTG13) with the closest known relatives [2, 3]. Initially, the infection targets the upper and lower respiratory tract, but virus can then travel to multiple different organs and infection can lead to multi-organ acute damage and post-acute sequelae [4–7].

In symptomatic individuals, common symptoms associated with COVID-19 are fever (greater than 38°C), cough, fatigue, headache, shortness of breath and chills. Symptoms can vary however, with over 20 different symptoms commonly reported by people with COVID-19 with factors such as vaccine status and the variant involved influencing the symptoms that are most common [8]. Risk of severe disease and death are most strongly correlated with age, with older people suffering particularly poorly. Other factors such as co-morbidities like obesity and diabetes are also clear risk factors for a worse outcome [9–12]. Despite these risk factors, vaccination has dramatically improved outcomes across populations and rates of deaths and severe disease in countries with high rates of vaccination have improved dramatically in the past year. Issues persist however, with deaths still occurring (albeit at a lower rate than pre-vaccination), transmission continuing despite vaccination, infection levels remaining high, and rates of long-term issues associated even with mild infections becoming more prominent. For example, a recent study in America showed that individuals previously infected with SARS-CoV-2 are at increased risk of incident cardiovascular disease spanning several categories, including cerebrovascular disorders, dysrhythmias, ischemic and non-ischemic heart disease, pericarditis, myocarditis, heart failure and thromboembolic disease [13].

The average latent period (time period between infection and becoming capable of infecting others) of SARS-CoV-2 infection is 3–6 days, while the average incubation period (time period between infection and the onset of symptoms) is 5–7 days with a range of between 2 and 14 days [14–17]. Interestingly, data suggests that the serial interval (time from illness onset in the primary case to illness onset in the secondary case), latent and incubation periods are variant dependent, with Omicron, for example, showing a shorter serial interval compared to the ancestral Wuhan strain [18–21]. Importantly, because the latent period tends to be shorter than the incubation period, spread of virus is possible pre-symptomatically, although cumulative data suggests that the secondary attack rate of pre-symptomatic individuals is lower than a symptomatic individual [22–25]This means however, that in a pandemic such as COVID-19, and potentially others in the future, a percentage of the population are circulating, infecting others and do not have symptoms. Recognising this and managing it is key to stopping rapid spread of the virus.

This study was designed to focus on asymptomatic people working in a healthcare setting in Turkey during a major wave of infection in December 2020. Between March and December 2020, there were 93,104 confirmed cases of SARS-CoV-2 infection in the Samsun province and 1,855 deaths, while in Turkey as a whole there were 2,208,652 cases and 20,881 deaths up to the end of December 2020 [26]. The objectives of the current study were to examine the burden of SARS-CoV-2 infection in a cohort of asymptomatic people, working in a tertiary level hospital before the vaccine rollout began (first vaccines were administered on 11^th^ January 2021). This study was conducted by (i) Screening blood samples for the presence of antibodies against SARS-CoV-2, and (ii) Screening nasal and throat swabs for the presence of SARS-CoV-2 RNA using reverse transcription quantitative polymerase chain reaction (RT-qPCR).

## Materials and methods

### Sampling

Approval and permission for this study was granted by the Clinical Research Ethics Board of The Samsun Training and Research hospital, reference number, GOKA/2021/4/10. Samples were collected from the 22^nd^ December 2020 to the 25^th^ December 2020.

This study was conducted in a pandemic hospital in Samsun, a tertiary healthcare centre with approximately 2600 healthcare personnel (HCP). Samsun is the biggest city on the northern coast of Turkey with a population of over one million and the hospital provides healthcare services to approximately 800,000 people every year. The current study was carried out on a voluntary basis. A convenience sampling approach was utilised whereby hospital workers were approached with information about the study and asked if they would like to participate. 176 HCWs including 17 physicians, 13 nurses, 22 technicians and 123 other (non-clinical) hospital staff e.g cleaners, security staff and front desk officers were enrolled on the study with a mean age of 39.56 ± 8. The volunteers were divided into three age groups: (i) <30 years old, (ii) 30 to 40 years old, and (iii) >40 years old (Table 1). All participants were asked whether they knew if they had been infected with SARS-CoV-2 previously, based on a test result or on the presence of symptoms associated with COVID-19 as defined by the WHO [26], and all stated no. All participants were also asked to describe how they were feeling at the time of sample collection and all participants reported to be feeling well without any symptoms associated with COVID-19 as defined by the WHO [27]. No individuals were vaccinated against COVID-19 as the vaccine had not yet been rolled out in Turkey at the time of the study.

**Table 1:** The demographic distribution of healthcare workers who voluntarily participated in the current study according to gender, job type and age.

Features	Number Tested (n)	Test Applied	
		RT-qPCR	eCLIA
**Total**	176	111	176
**Gender**			
Female	90	57	90
Male	86	54	86
**Age groups**			
<30	26	18	26
30–40	65	41	65
>40	85	52	85
**Job**			
Physician	17	14	17
Nurse	13	6	13
Technician	22	17	22
Others*	124	74	124

*Other hospital staff: e.g desk officer, security, cleaners.

### Antibody screening against SARS-CoV-2

To detect both IgM and IgG antibodies against SARS-CoV-2, serum samples were taken from 176 healthcare volunteers (111 of which were also swabbed for RT-qPCR) and were tested by electrochemiluminescence immunoassay (eCLIA) (Lifotronic, China) on an automated ECL immunoassay analyser (eCL8000) according to the manufacturer’s instructions. The analyser used the positive and negative reference controls provided with the kit to determine cutoff points and subsequently, the sample results were expressed in terms of reactivity or non-reactivity using the pre-determined cutoff value index (COI: sample signal value/cutoff value). A COI of < 0.8 was interpreted as non-reactive, while a COI of ≥ 1.2 was considered reactive and a COI of great than or equal to08 but below1.2 was accepted as indeterminate. The clinical sensitivity of the assay for both IgG and IgM is reported as 92.5% and the specificity is 94%.

### SARS-Cov-2 Double gene RT-qPCR

Nasal and throat swabs were performed on 111 of the 176 volunteers. RNA extraction and RT-qPCR were conducted using the Bio-speedy® COVID-19 qPCR detection kit, Version 1 (Bioexen, Turkey) with primers and probes targeting parts of the SARS-CoV-2 N and Orf1ab genes. The PCR was carried out in a Rotorgene (Qiagen, Germany) and the manufacturer’s instructions were always followed. The PCR reaction was carried out in a total volume of 20 μL and consisted of 5 μL of Oligo Mix, 10 μL of 2X Prime Script Mix, and 5 μL of RNA template. The RT-qPCR conditions were 5 minutes at 52°C, 10 seconds at 95°C and 40 cycles of 1 second at 95°C and 30 seconds at 55°C. Each run included a positive and negative control as well as an internal control (IC) targeting the Human RNase P gene.

Ct values of less than 38 were classified as a positive result. The analytical and clinical performance of the kit was determined by the Turkish Ministry of Health, General Directorate of Public Health, Department of Microbiology Reference Laboratories and Biological Products (HSGM). The analytical sensitivity of the kit is reported to be 99.4% and its specificity is reported to be 99.0%. The limit of detection for this kit is reported by the manufacturer as being approximately 500 genome copies per ml.

### Statistical analysis

Statistical analyses were mostly conducted using SPSS software version 22.0 (IBM, Armonk, NY, USA) and P <0.05 value was accepted to be statistically significant. Categorical variables were expressed as frequency and percentage and were compared using the chi-square test. The 95% confidence intervals for apparent prevalence were not calculated using SPSS. Instead, they were calculated using Epitools (https://epitools.ausvet.com.au/) with the Clopper Pearson exact method.

## Results

### Antibody profiling

Of the 176 people sampled, 26 were less than 30 years-old, 65 were from 30 to 40 years-old and 85 were above the age of 40. Of these, 52 (29.55%) had antibodies against SARS-CoV-2 present in their serum sample. As detailed in Table 2, 13 (7.39%) out of 176 samples were positive for the presence of IgM antibodies against SARS-CoV-2 only while 12 (6.82%) out of 176 samples were positive for IgG antibodies against SARS-CoV-2 only. Furthermore, 27 (15.34%) samples were positive for both IgM and IgG together. There was a significant difference for overall anti-IgM, anti-IgG, and both-antibody responses (p<0.001). In terms of job type, 17.65% (3 / 17) of physicians, 23.08% (3 / 13) of nurses, 22.73% (5 / 22) of technicians and 33.07% (41 / 124) of other hospital staff were positive for anti-SARS-CoV-2 antibodies. There was no significant association between likelihood of being positive for SARS-CoV-2 antibodies and the type of job a person held.

**Table 2:** The results of electrochemiluminescence immunoassay in tested HCWs according to gender, age groups, and job type.

Features	Tested n (%)	Number (n)%; 95% confidence interval			*p value*
		IgM	IgG	*IgM+IgG*	
**Total**	176 (100)	13 / 176 (7.4%; 3.9–12.3%)	12 / 176 (6.8%; 3.6–11.6%)	27 / 176 (15.3%; 10.4–21.5%)	<0.001
**Gender**					
Female	90 / 176 (51.1%)	3 / 90 (3.3%; 0.7–9.4%)	4 / 90 (4.4%; 1.2–11%)	14 / 90 (15.6%; 8.8–2.5%)	>0.05
Male	86 / 176 (48.86%)	10 / 86 (11.6%; 5.7–20.4%)	8 / 86 (9.3%; 4.1–17.5%)	13 / 86 (15.1%; 8.3–24.5%)	
**Ages**					
<30	26 / 176 (14.8%)	0	3 / 26 (11.5%; 2.5–30%)	5 / 26 (19.2%; 6.6–39.4%)	>0.05
30–40	65 / 176 (36.9%)	7 / 65 (10.8%; 4.4–20.9%)	6 / 65 (9.2%; 3.5–19%)	7 / 65 (10.8%; 4.4–20.9%)	
>40	85 / 176 (48.3%)	6 / 85 (7.1%; 2.6–14.7%)	3 / 85 (3.5%; 0.7–10%)	15 / 85 (17.7%; 10.2–27.4%)	
**Jobs**					
Physician	17 / 176 (9.7%)	1 / 17 (5.9%; .02–28.7%)	0	2 / 17 (11.8%; 1.5–36.4%)	>0.05
Nurse	13 / 176 (7.4%)	1 / 13 (7.7%; 0.2–36%)	0	2 / 13 (15.4%; 1.9–45.5%)	
Technician	22 / 176 (12.5%)	2 / 22 (9.1%; 1.1–29.2%)	0	3 / 22 (13.6%; 2.9–35%)	
Others*	124 / 176 (70.5%)	9 / 124 (7.3%; 3.4–13.3%)	12 / 124 (9.7%; 5.1–16.3%)	20 / 124 (16.1%; 10–23.8%)	

*Other hospital staffs. e.g desk officer, security, cleaners.

Of the 13 HCWs who were found to be anti-SARS-CoV-2 IgM positive (IgM only), 3 were female and 10 were male. Of this group, 7 were in the 30–40-year-old age group, 6 were in the over 40-year-old group. No one less than 30 years old was positive for only IgM antibodies against SARS-CoV-2. Of the 12 IgG seropositive volunteers (IgG only), 4 were female and 8 were male. The distribution by age of IgG seropositivity was: 3 people less than 30-year-olds, 6 people between 30 and 40 years old, and 3 people over the age of 40 years old. Of the 27 HCWs who were positive for both IgM and IgG antibodies against SARS-CoV-2, 5 were below the age of 30 years-old, 7 were in the 30–40 years-old age group and 15 were above the age of 40 years-old. No statistically significant difference was measurable between the different age groups.

### Testing for SARS-CoV-2 RNA by RT-qPCR

All results associated with the RT-qPCR analysis are detailed in Table 3. From the 176 volunteers that donated blood for the antibody study, 111 were also swabbed for PCR. Of those 111, 33 (29.72%) were positive for the presence of SARS-CoV-2 RNA and the Ct values of the PCRs were all between 18 and 25. In terms of age group, 6 positives were below the age of 30 years old, 10 were between 30 years old and 40 years old, and 17 were greater than 40 years old. There was no significant association between age group or gender and PCR positivity.

**Table 3:** RT-qPCR results of tested voluntarily HCWs according to gender, age groups, and jobs.

Features	RT-qPCR tested	Positives	*p* value
		n (%); 95% confidence interval	
	n (%)		
**Total**	111 (100)	33 / 111 (29.7%; 21.4–39.2%)	
Female	57 / 111 (51.35%)	17 / 57 (29.8%; 18.4–43.4%)	>0.05
Male	54 / 111 (48.65%)	16 / 54 (29.63%; 18–43.6%)	
**Ages**			
<30	18 / 111 (16.22%)	6 / 18 (33.33%; 13.3–59%)	>0.05
30–40	41 / 111 (36.94%)	10 / 41 (24.39%; 12.4–40%)	
>40	52 / 111 (46.85%)	17 / 52 (32.69%; 20.3–47.1%)	>0.05
**Jobs**			
Physician	14 / 111 (12.61%)	4 / 14 (28.57%; 8.4–58.1%)	>0.05
Nurse	6 / 111 (5.41%)	1 / 6 (16.67%; 0.4–64%)	
Technician	17 / 111 (15.32%)	6 / 17 (35.29%; 14.2–61.7%)	>0.05
Others*	74 / 111 (66.67%)	22 / 74 (29.73%; 19.7–41.5%)	

*Other hospital staffs. e.g desk officer, security, cleaners.

Similarly, when assessing results according to job type it was found that 4 / 14 doctors (28.57%), 1 / 6 nurses (16.67%), 6 / 17 technicians (35.29%) and 22 / 74 people in other hospital-based jobs (29.73%) were positive for SARS-CoV-2 RNA. Again, there was no significant association identified between job type and RNA positivity.

Finally, of the 33 people positive for SARS-CoV-2 RNA by PCR, 26 were also antibody positive, with no antibody detectable in 7 PCR positive volunteers. 18 of this group of 26 were positive for both IgM and IgG, 3 were only positive for IgM and 5 were only positive for IgG antibodies against SARS-CoV-2.

## Discussion

At the beginning of the pandemic, it was perceived by many that only those with symptoms could spread the virus. However, over time, it became obvious that asymptomatic or presymptomatic individuals had the ability to spread the virus too [28–30]. This creates a control challenge, as it is difficult to identify infectious individuals before they spread the virus to others, and this is a particularly critical issue in environments such as care homes or hospitals where vulnerable people are more commonly located [31–33].

This study was designed to determine the level of prior and current infection in a cohort of workers in a hospital that dealt with COVID-19 patients. A study carried out across 32 hospitals in 7 regions of Turkey following the first wave of SARS-CoV-2 infection in early 2020 showed a seroprevalence of 6.1% while 68% of those that were positive stated that they did not know they had been previously infected [34]. In a similar but smaller study carried out in May 2020, focused on workers in 3 hospitals in Istanbul and Kocaeli, seropositivity ranged from 1% - 6.4%, with job type apparently influencing likelihood of seropositivity[35]. Based on these publications and the fact that this study was carried out in the middle of a major wave of infection, we estimated an incidence rate of 12.5% in our population in advance of the study taking place, providing a target sample size of 174 with 85% power. For the study we recruited a total of 176 asymptomatic volunteers from the healthcare setting and tested them for the presence of SARS-CoV-2 antibodies. We also tested 111 of that 176 for the presence of SARS-CoV-2 RNA (by nasal and throat swab). We detected the presence of both SARS-CoV-2 IgG and / or IgM antibodies in 52 of the 176 volunteers (29.6%). This higher-than-expected seroprevalence is likely a reflection of the timing of collection which was during the second major wave of infection in Turkey while other studies in Turkey were all carried out prior to the second wave. A trend of increased seroprevalence between early 2020 and late 2020 has been reported by a number of studies from other parts of the world. In the Netherlands a report noted a seroprevalence increase from 4.1% to 13.8% in a paediatric population when sampling during the first or second waves of infection in 2020 [36]. In a cohort of healthcare worker in Belgium, seroprevalence increased by 29.4% (12.4% to 41.8%) while in a healthcare population in Poland seroprevalence increased from 2.4% to 22.9% from one wave to the next in 2020 [37, 38].

Studies have indicated that in response to a SARS-CoV-2 infection both IgG and IgM antibody titres generally increase rapidly and start to become detectable 5–7 days post-symptom onset (and possibly earlier), with IgM detection slightly preceding or rising concurrently with detectable IgG [39, 40]. Titres of IgM antibodies decrease rapidly to baseline after approximately 12 weeks while IgG antibodies can persist for 6 months or possibly more [41, 42]. Volunteers that were IgM positive and IgG negative were likely to have been infected very recently and may still have been shedding virus when sampled, despite a lack of symptoms. Volunteers that were IgM and IgG positive were also probably infected in the recent past, while those that were IgM negative and IgG positive were probably infected in the distant past, (beyond 3 months). There is no direct association between antibody presence or absence and infectiousness, so it is unclear whether these individuals were shedding virus but it is likely that some were and could therefore have infected others.

When considering seroprevalence, it is important to acknowledge that with all tests there is a level of false-negatives and false-positives that may be present, and this should add to our interpretation [43]. Based on clinical validation studies by the manufacturer, sensitivity and specificity of the tests were above 90% but this leaves room for error. When interpreting this data, it is also important to note that samples were collected during a surge of infection which began in early November 2020 and peaked in early December and reduced slowly through December and early January [26]. This means that prevalence was high during the sampling period which reduces the likelihood of false positives.

At certain stages of the pandemic cases tended to rise in different age groups unevenly. For example, as the pandemic progressed in Ireland case numbers generally rose in individuals below the age of 45 before rising in older cohorts as a new wave of infection hit the country [44] In this study, there was no statistically significant association between antibody positivity and gender, age group or job within the hospital. Previously, a study conducted in HCWs in Italy showed that IgM seroprevalence was higher in all age groups than IgG positivity [45], while other studies have shown a relationship between age and IgG titre [46, 47]. This data is still evolving and is likely to be associated with disease severity and time of sampling post-infection as well as other factors such as individual behaviour and knowledge of the virus transmission routes. It is also important to note that the number of volunteers in each age cohort of this study is not equal and numbers are relatively low so selection bias cannot be ruled out thus making it difficult to draw strong conclusions on these small differences in percentage seroprevalences at this time.

Alongside antibody seroprevalence, RT-qPCR to detect the presence of SARS-CoV-2 RNA was used on a sub-section of the volunteers. Of the 111 people swabbed, 33 were positive by nasal and throat swab for the presence of SARS-CoV-2 RNA. Of these 33, 26 were also positive by antibody ELISA, while antibody against SARS-CoV-2 could not be detected in 7 people’s samples. Non-detection of antibodies could indicate that the infection was very recent, and antibodies had not been produced yet or antibodies were produced but at a very low level and so were not detectable. These non-overlapping positives supports data that suggests that a combination approach to diagnosis, using more than one test on a person improves the likelihood of a diagnosis [48, 49]. While this is not practical for population wide testing, in targeted situation, such as persistent infections or potential Long COVID cases, a combination of diagnostic tests is supported. The level of antibody production in PCR positive people is also relevant to the potential development of more severe outcomes as recent studies have shown a positive association between a rapid immune response, an earlier resolution of disease and reduced likelihood of Long COVID [5, 42, 47, 50–52].

It has been frequently reported that people can be positive by PCR for weeks post-infection, but they are unlikely to be shedding significant amounts of infectious virus beyond about 10–15 days post—infection, particularly those that are asymptomatic [18, 53, 54]. In this study 5 individuals were PCR positive (Ct below 25) for SARS-CoV-2 and IgG positive but IgM negative. One must always consider the possibility of false negatives in these scenarios, but if true, it could suggest persistent shedding of SARS-CoV-2 RNA for extended periods beyond the production of IgM antibodies. Although infectious virus was not recovered from nasal swabs, persistent detection of RNA for extended periods, particularly with low (<25) PCR Ct values does raise questions around the potential persistence of virus in areas of the body apart from the nasal passage or throat [55–58]. This raises the further possibility that individuals may remain infectious for extended periods of time. More work is required to understand the temporal and spatial dynamics of SARS-CoV-2 in individuals where this might occur.

All volunteers stated that they had not recently experienced symptoms commonly associated with COVID-19 such as a fever, sore throat, loss of taste or smell, coughing etc. These individuals were either asymptomatically infected, in a presymptomatic state or infected with very mild symptoms. A limitation of this study is that no follow-up was carried out, so it is not known whether individuals developed symptoms post sampling. In any case, symptoms are not required to spread SARS-CoV-2 to others [28, 59–63]. Rapid spread of SARS-CoV-2 between people in multiple environments including hospitals, creates major control difficulties and exposes almost everyone to the risk of infection. This is particularly important in locations that have high concentrations of particularly vulnerable people. A limitation of this study is that we do not know which units’ individuals worked in or how these individuals interacted with each other inside or outside of work. Because of this we cannot say whether parts of the hospital were associated with higher rates of SARS-CoV-2 positivity and also whether these areas were contributing to virus spread. Previous studies have indicated that rather than particular areas of the hospital being associated with higher risk of infection, it is the activity of the individual which is the most important risk factor; workers dealing directly with SARS-CoV-2 positive patients, regardless of role, are at increased risk of infection. Ventilation and PPE use as well as the general prevalence of infection in the population are also factors that influence risk of infection both in hospitals and other locations [64–70].

This study adds to other studies highlighting the fact that large proportions of populations were infected unknowingly and were potentially spreading the virus to others during a period when there was no vaccine or treatment available. Reliance on symptoms to limit the movement of people, which formed the cornerstone of many control approaches across the world, will always fail to restrict the cohort of the population that is infected asymptomatically. The employment of effective strategies to identify infected / infectious people without relying on symptoms will have to form the cornerstone of public health responses to future pandemics and may help to avoid total lockdowns as occurred in many countries globally.
